# Strength or Motor Control: What Matters in High-Functioning Stroke?

**DOI:** 10.3389/fneur.2018.01160

**Published:** 2019-01-09

**Authors:** Neha Lodha, Prakruti Patel, Agostina Casamento-Moran, Emily Hays, Sharon N. Poisson, Evangelos A. Christou

**Affiliations:** ^1^Department of Health and Exercise Science, Colorado State University, Fort Collins, CO, United States; ^2^Department of Applied Physiology and Kinesiology, University of Florida, Gainesville, FL, United States; ^3^Department of Neurology, University of Colorado, Aurora, CO, United States

**Keywords:** ankle movement, weakness, motor impairments, lower limb, walking, driving, functional mobility, hemiparesis

## Abstract

**Background:** The two primary motor impairments that hinder function after stroke are declines in strength and motor control. The impact of motor impairments on functional capacity may vary with the severity of stroke motor impairments. In this study, we focus on high-functioning stroke individuals who experience mild to moderate motor impairments and often resume prior activities or return to work. These tasks require the ability to move independently, placing high demands on their functional mobility. Therefore, the purpose of this study was to quantify impairments in strength and motor control and their contribution to functional mobility in high-functioning stroke.

**Methods:**Twenty-one high-functioning stroke individuals (Fugl Meyer Lower Extremity Score = 28.67 ± 4.85; Functional Activity Index = 28.47 ± 7.04) and 21 age-matched healthy controls participated in this study. To examine motor impairments in strength and motor control, participants performed the following tasks with the paretic ankle (1) maximum voluntary contractions (MVC) and (2) visuomotor tracking of a sinusoidal trajectory. Strength was quantified as the maximum force produced during ankle plantarflexion and dorsiflexion. Motor control was quantified as (a) the accuracy and (b) variability of ankle movement during the visuomotor tracking task. For functional mobility, participants performed (1) overground walking for 7 meters and (2) simulated driving task. Functional mobility was determined by walking speed, stride length variability, and braking reaction time.

**Results:** Compared with the controls, the stroke group showed decreased plantarflexion strength, decreased accuracy, and increased variability of ankle movement. In addition, the stroke group demonstrated decreased walking speed, increased stride length variability, and increased braking reaction time. The multiple-linear regression model revealed that motor accuracy was a significant predictor of the walking speed and braking reaction time. Further, motor variability was a significant predictor of stride length variability. Finally, the dorsiflexion or plantarflexion strength did not predict walking speed, stride length variability or braking reaction time.

**Conclusions:** The impairments in motor control but not strength predict functional deficits in walking and driving in high-functioning stroke individuals. Therefore, rehabilitation interventions assessing and improving motor control will potentially enhance functional outcomes in high-functioning stroke survivors.

## Introduction

The two primary motor impairments that hinder function after stroke are declines in strength and motor control (1–3). The decline in strength manifests as muscle weakness such that individuals with stroke experience difficulty in generating maximal forces with the paretic limb (4, 5). The decline in motor control manifests as an impaired ability to produce precise and steady motor output with the paretic limb (6, 7). Decrease in strength and motor control have independently been shown to contribute to impaired function in stroke (8, 9).

The decline in paretic leg strength is associated with deterioration in lower-limb function. For example, decreased paretic knee strength is related to increased postural sway (10). Similarly, reduced paretic knee extensor strength is linked to slower self-selected walking speed and impaired stair-climbing ability (11, 12). Despite extensive research on the association between strength and motor function in stroke survivors, the evidence regarding role of impaired motor control on functional capacity is sparse, especially for the lower-limb (13, 14). A study on the upper-limb motor control demonstrated that increased variability and reduced accuracy for finger forces was related to poor functional outcome during grasp and release in stroke (15). The influence of strength and motor control on functional capacity potentially varies with the severity of stroke motor impairments. Buchner et. al. argued that functional status of older individuals is critical for determining the potential for improvement in motor function after strength training (16). Therefore, an important but unaddressed question in the stroke literature is whether the contribution of strength and motor control to functional capacity depends on the severity of stroke motor impairments.

In the current study, we focus on high-functioning stroke individuals with mild to moderately severe motor impairments. A compelling reason to study high-functioning stroke is that a large proportion of individuals experience mild-moderate motor impairments (17, 18). Others with initially severe motor impairments experience spontaneous motor recovery and convert to less severe motor impairment (19, 20). Individuals with mild or moderate motor impairments are considered high-functioning and typically resume prior activities, return to work and often live on their own (21, 22). These tasks require the ability to move independently, placing high demands on functional mobility (e.g., walking and driving). Therefore, a key focus of the current study is to examine the extent to which the impairments in strength and motor control influence functional mobility in high-functioning stroke individuals.

We investigate two key forms of functional mobility in high-functioning stroke individuals: overground walking and driving. Overground walking facilitates the study of natural walking pattern. We quantify overground walking function with walking speed and stride length variability. Walking speed is frequently used as a rehabilitation outcome and an indicator of functional independence post-stroke (23–25). Stride length variability is linked to the loss of balance and increased risk of falls in older adults (26–28). We study driving in a simulated environment. Simulated driving provides a good approximation of on-road driving function in a safe and controlled laboratory environment (29). We quantify driving function with braking reaction time. Extensive data from driving research suggests that braking reaction time is a strong predictor of crash risk and the overall driving ability (30, 31). Together, walking speed, stride length variability, and braking reaction time will provide insight into key elements of functional mobility in high-functioning stroke survivors.

The purpose of the current study was to quantify impairments in strength and motor control and their contribution to overground walking and simulated driving in high-functioning stroke individuals. Specifically, we examined isometric ankle strength, and motor control i.e., accuracy and variability of ankle position in dorsiflexion-plantarflexion. We applied stringent clinical criteria based on activity level and severity of motor impairments to select individuals with high-functioning status. We hypothesized that compared with matched healthy controls, high-functioning stroke individuals will demonstrate impaired strength and motor control that will contribute to deficits in overground walking and driving function.

## Methods

### Participants

Twenty-one high-functioning stroke individuals (65.04 ± 13.72 years) and 21 healthy age-matched controls (68.59 ± 8.53 years) participated in the study. The participant characteristics for both the groups are presented in Table 1. The inclusion criteria for high-functioning stroke participants were: (1) diagnosed with a unilateral cerebrovascular accident at least 9 months prior to testing, (2) a minimum of 15 degrees of ankle dorsiflexion and 5 degrees of active plantarflexion, without assistance, (3) ability to walk 3 min without assistance, (4) ability to grasp the steering wheel, (5) ability to understand and follow a three step command, and (6) meet high-functioning status.

**Table 1:** Participant characteristics.

	**Stroke (*N* = 21)**	**Control (*N* = 21)**
Age (years)	65.04 ± 13.72	68.59 ± 8.53
Sex (Male/Female), *N*	13 / 8	11 / 10
Height (cm)	171.78 ± 9.55	170.89 ± 10.07
Hemiparetic side (left/right), *N*	4 / 17	n/a
Time since stroke (years)	4.79 ± 4.66	n/a
**LESION LOCATION**		
Cortical	10	n/a
Subcortical	4	n/a
Unknown	7	n/a
FMA	27.61 ± 4.85	n/a
FAI	28.47 ± 7.04	31.57 ± 4.66

*FMA, Fugl-Meyer Assessment of Lower Extremity (max score 34); FAI, Frenchay Activity Index (max score 45); n/a, not applicable. All scores are mean ± standard deviation*.

To qualify as high-functioning, individuals with stroke met at least one of the two distinct criteria including (a) mild to moderate lower extremity motor impairment i.e., Fugl-Meyer Lower-extremity Assessment score (FMA) >16/32 (32) or (b) moderate activity level i.e., Frenchay Activity Index (FAI) >16/45 (33, 34). Exclusion criteria for all participants were the presence of any other neurological or musculoskeletal deficits, visual neglect, uncorrected vision, or hearing impairments, pain, and predisposition to motion sickness in simulated driving environment. Prior to participation, all individuals read and signed an informed consent approved by the University of Florida's Institutional Review Board.

### Experimental Procedures

The experimental session lasted ~3 h. During the session, each participant performed clinical evaluations and four experimental tasks that included: (i) maximum voluntary contractions (MVC) of ankle dorsiflexion and plantarflexion,(ii) visuomotor tracking task with isolated ankle movements, (iii) overground walking, and (iv) driving in a simulated environment.

#### Clinical Evaluations

For the stroke group, the degree of motor impairments in leg and foot was assessed using the lower extremity subsection of Fugl-Meyer Motor Assessment (35). FMA is most widely used measure of motor impairment in stroke that examines volitional control of limbs. For all participants, the activity level was determined by Frenchay Activity Index (36). FAI assesses a broad range of activities associated with everyday life such as doing laundry, shopping, engagement in social occasions, and gainful work.

#### Maximum Voluntary Contraction

##### Experimental set-up

The participants were seated comfortably in an upright position with hip joint at ~90° flexion, the knee at ~90° flexion, and the ankle in a neutral position. In line with clinical recommendations (37, 38) and previous work (39–41) examining ankle strength in stroke, we chose the seated position for MVC measurement. Specific instructions were given to exert force at the ankle without engaging or moving the hip, knee, or the trunk while maintaining a stable posture until trial completion. The experimenter monitored the posture of the participant to limit extraneous force production and ensure compliance with the instructions.

##### Task

The maximal isometric force was measured during ankle plantarflexion and dorsiflexion. Participants were instructed to exert maximum force for 3 s. The stroke group used the paretic leg and the control group used the non-dominant leg to perform MVC task. The participants completed three to five MVC trials or until three MVC trials were within 5% of each other. To minimize fatigue we provided a rest period of 60 s between trials. The MVC task order for the ankle plantarflexion and dorsiflexion was randomized between participants.

#### Visuomotor Tracking Task

##### Experimental set-up

**Figure 1:**
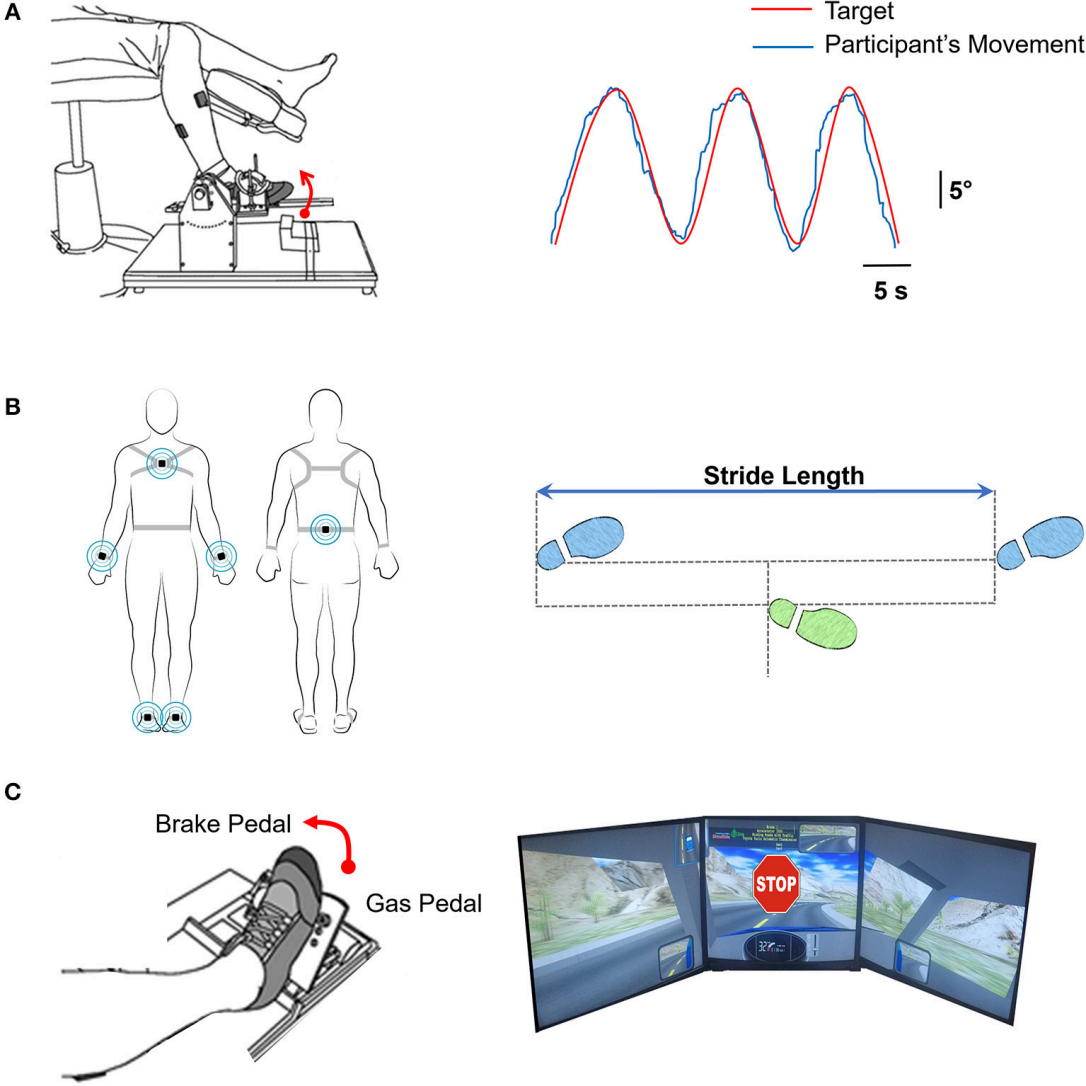
**(A)** Motor control. Left: Participants tracked a trajectory on the computer with ankle dorsiflexion and plantarflexion movements. Right: The computer feedback provided to the participants of tracking a sinusoidal target (red line; at 0.3 Hz through a 20° range of motion) with the ankle movement (blue line). **(B)** Overground walking. Left: Participants were instrumented with six inertial movement sensors on their wrist (2), ankle (2), sternum, and lumbar spine (*Recreated with permission from APDM Mobility Lab Inc., Portland, Oregon, USA*). Participants were instructed to walk at their natural and comfortable pace for 7 m overground. We measured the walking speed and stride-length variability **(C)** Driving in a simulator Left: The participants were instructed to drive on a winding road for 3 min. At random times during the driving course, an unexpected STOP stimulus was presented. Participants were asked to respond as fast as possible by pressing the brake pedal. Right: Visual feedback of the driving scenario with the sudden stop stimulus.

The participants were seated comfortably in an upright position in front of a 32-inch monitor (Sync Master™ 275t+, Samsung Electronics America, NJ, USA) located 1.25 m away at the eye level. The monitor provided the visual feedback of the movements produced by the ankle. Participants affirmed that they could clearly see the visual display. The hip joint was flexed to ~90° with 10° abduction, the knee was flexed to ~90°, and the ankle was in a neutral position. The stroke group used the paretic ankle and the control group used the non-dominant ankle to perform the visuomotor tracking task. The foot rested on a custom device with an adjustable foot plate and was secured by straps over the metatarsals to ensure a secure position and simultaneous movement between the device and the foot. The axis of rotation of the custom device was positioned in line with the axis of rotation of the ankle. Figure 1A shows a schematic representation of the experimental set-up.

##### Task

The participants tracked a sinusoidal target at a frequency of 0.3 Hz using isolated ankle joint movement. The targeted movement ranged from 5° ankle plantarflexion to 15° ankle dorsiflexion. The participants were instructed to track the sinusoidal target (red line) as accurately as possible by moving their ankle up and down. The participants received visual feedback of their performance (blue line) (Figure 1A). Each trial lasted ~ 35 s. We provided a rest period of 30 s between consecutive trials to minimize fatigue. Participants performed 3 practice and 5 test trials (i.e., 8 total trials). One stroke participant failed to complete the visuomotor tracking task and was excluded from the analysis.

#### Overground Walking

##### Experimental set-up and task

We placed a total of six wearable sensors (APDM Mobility Lab Inc., Portland, Oregon, USA) on the wrists, ankles, sternum, and lumbar spine of the participants to examine the kinematics of overground walking. Participants were instructed to walk a 7 m distance at their natural and comfortable pace. Three trials were performed with a 90 s rest period between trials. Figure 1B shows the schematic representation of a participant with the wearable sensors.

#### Simulator Driving Task

##### Experimental set-up

Participants were seated in an upright position in a professional driving simulator seat (AplusB software, Myrtle Beach, South Carolina, 7 USA), with a steering wheel, a gas pedal, and a brake pedal. The driving environment was presented on three 24-inch computer monitors located side by side at eye level. The simulated driving environment included driving a compact car on a winding road with oncoming traffic on a clear sunny day. Figure 1C shows the schematic representation of participant's foot position and simulator driving environment.

##### Task

Participants were instructed to drive at 30 km/h in the center of the lane for 3 min. At random times during the driving course, an unexpected STOP stimulus was presented. The participants were instructed to respond as quickly as possible to the STOP stimulus by releasing the gas pedal and pressing the brake pedal. A total of 10 STOP stimuli were presented within a 3 min driving period. A short 20 s trial of driving familiarization preceded the test trial. The stroke group used the paretic leg and the control group used the dominant leg to perform the simulated driving task. Two stroke participants failed to complete the simulated driving task and were excluded from the analysis.

### Data Measurement and Analysis

#### Ankle Force Measurement

The MVC was measured using a force transducer (Model 41BN, Honeywell, Morristown, NJ, USA) located parallel to the force direction on a customized foot device. The force signals were band-pass filtered from 0.03 to 20 Hz, amplified by a gain factor of 50 (Bridge-8 world precision instrument Inc., FL, USA), sampled at 1000 Hz (NI-DAQ card, Model USB6210, National Instruments, Austin, TX, USA), and stored on a research workstation for offline analysis. *Strength:* We quantified strength with the MVC of ankle dorsiflexion and plantarflexion. We defined the MVC as the highest MVC obtained among the three trials.

#### Ankle Position Measurement

The ankle position during the visuomotor tracking task was measured using a low-friction potentiometer (SP22G-5K, Mouser Electronics, Mansfield, TX, USA) located directly lateral to the fibular malleolus. The position signals were sampled at 1000 Hz (NI-DAQ card, Model USB6210, National Instruments, Austin, TX, USA) and saved for offline analysis. A custom routine written in Matlab® (Math Works™ Inc., Natick, Massachusetts, USA) controlled the visual presentation of each trial. *Motor Control:* We quantified motor control with the accuracy and variability of ankle movements averaged across 3 trials. The first 10 s and final 5 s of position data were eliminated from all analyses to account for initial position adjustments and early movement cessation caused by the anticipation of trial completion. Data were analyzed offline using a custom-written program in Matlab^®^ (Math Works™ Inc., Natick, Massachusetts, USA).

##### Accuracy

The accuracy of ankle position was measured by root mean squared error (RMSE) that quantifies the distance between the target and participant's position. The RMSE was calculated as

$$RMSE=\sqrt{\frac{\sum_{i=1}^{n}(X_{participant,\;i}-\;X_{target,\;i}{)}^{2}}{\text{n}}}$$

##### Variability

The variability of ankle position was quantified by standard deviation (SD) of ankle position within each trial. The movement signal was band-pass filtered between 0.2 and 0.4 Hz to remove the task-related frequency (sinusoidal target at 0.3 Hz). We quantified movement variability with the SD of the detrended position signal.

#### Overground Walking Measurement

Position and acceleration data from the movement monitors were used to detect gait events. The data were validated at the end of each trial and stored for offline analysis. *Walking Speed:* We computed the overground walking speed with the average speed across 3 trials. *Stride length variability*: We quantified the overground walking ability with stride length variability averaged across 3 trials. The magnitude of stride length variability over a single walking trial was determined as the coefficient of variation of the stride length (coefficient of variation = standard deviation of stride length/mean stride length × 100) averaged across both legs.

#### Simulator Driving Measurements

AplusB software was used to determine the braking reaction times. *Braking Reaction Time*: We measured braking reaction times as the time between presentation of STOP stimulus and application of brake pedal. Application of brake pedal was marked by a 10% change in brake pedal position from neutral position. We averaged the braking reaction time across 10 trials.

### Statistical Analysis

We compared the stroke and control groups using the independent *t*-test on the following measures: (i) strength (MVC for plantarflexion and dorsiflexion), (ii) motor control (RMSE and SD of ankle movement), (iii) overground walking speed, (iv) stride length variability, and (v) braking reaction time. To determine the relationship between strength, motor control, overground walking, and driving function, we used the Pearson's bivariate correlations.

To determine the contribution of strength and motor control to walking and driving function, we used a backward, multiple-linear regression model. The regression analysis included MVC plantarflexion, MVC dorsiflexion, RMSE, and SD of ankle position as the predictor (independent) variables. We ran separate regression models to predict walking speed, stride length variability, and braking reaction time (criterion/dependent, variables). The squared multiple correlation coefficient (*R*^2^) and the adjusted squared multiple correlation coefficient (adjusted *R*^2^) determined the goodness-of-fit of the model. *R*^2^ indicates the robustness of the linear combination of the variables predicting the criterion variables. The adjusted *R*^2^ accounts for *R*^2^'s overestimate of the percentage of the variance in the criterion variable that can be explained by the linear combination of the predictor variables, especially when the sample size is small and the number of predictors is large. In addition, we chose a model with the least number of predictors that demonstrated a significant adjusted *R*^2^. Statistical analyses were conducted with alpha level was set at 0.05 using the IBM SPSS Statistics 24.0 statistical package.

## Results

### Clinical Evaluations

The mean lower-extremity FMA score was 27.61 ± 4.85 (range 16–34) for the stroke group. The FAI score was 28.47 ± 7.04 (range 16–38) for the stroke group and 31.57 ± 4.66 (range 21–42) for the control group. No significant difference was found between the stroke and control group (|t40| = −1.51; *p* > 0.05) on the FAI score Thus, the FMA and FAI scores confirmed the stroke group to be high-functioning.

### Strength

**Figure 2:**
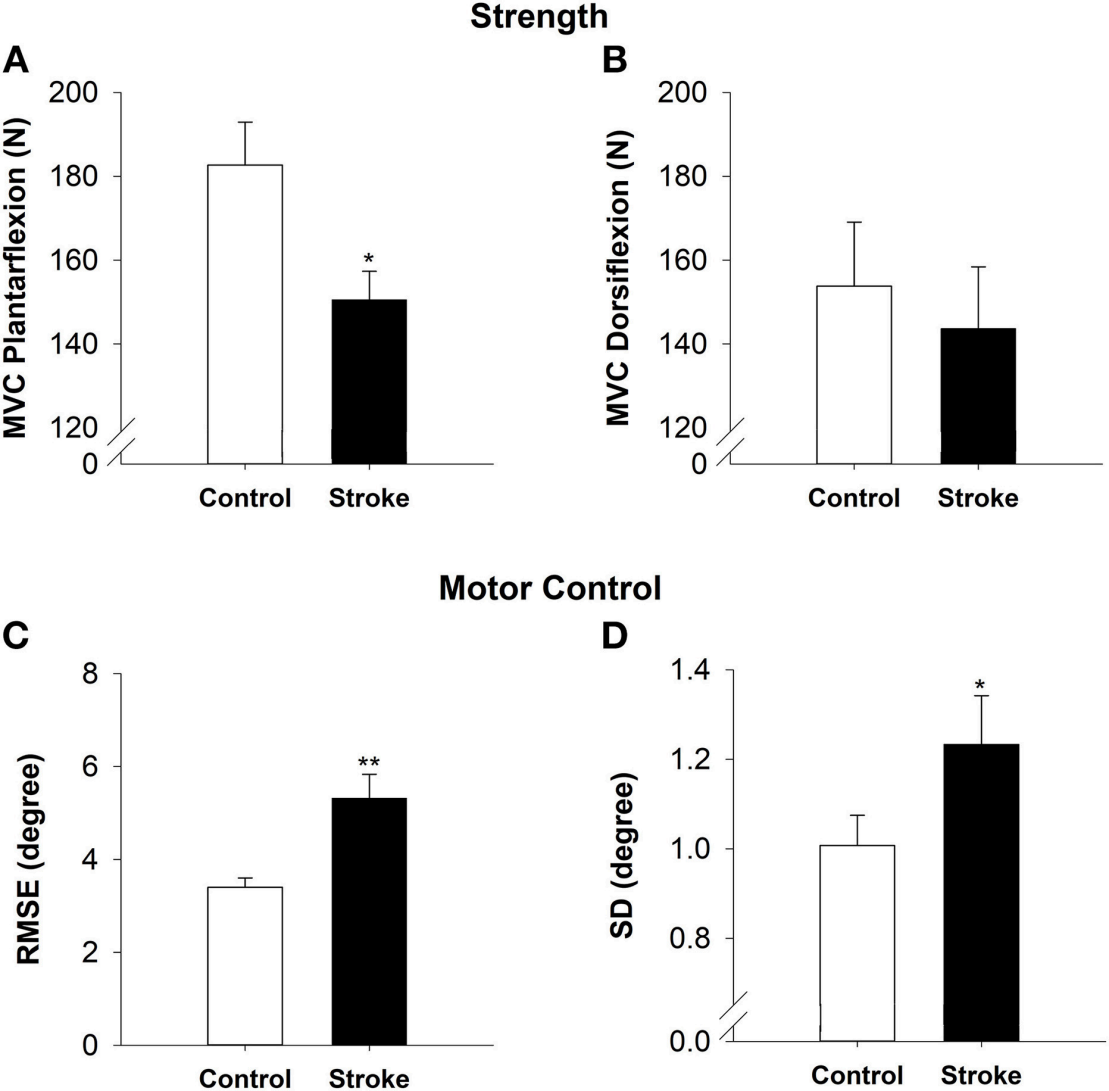
Strength and motor control in high-functioning stroke. Strength was quantified by MVC for plantarflexion **(A)** and dorsiflexion **(B)**. The high-functioning stroke group had significantly lower plantarflexion MVC than the control group. The dorsiflexion strength was not significantly different between groups. Motor control was quantified by accuracy **(C)** and variability **(D)** of ankle movement. The high-functioning stroke group had significantly greater RMSE and SD than the control group. Overall, high-functioning stroke participants demonstrated impaired strength (plantarflexion) and motor control compared with the healthy controls. *p < 0.05; **p < 0.01.

The high-functioning stroke group had decreased plantarflexion strength as compared with the controls (|*t*_40_| = −2.58; *p* < 0.05; Figure 2A). The plantarflexion MVC was 150.50 ± 30.52 N for the stroke group and 182.69 ± 46.94 N for the control group. There was no significant difference between the groups for dorsiflexion strength (|*t*_40_| = −0.47; *p* > 0.05; Figure 2B). The dorsiflexion MVC was 143.64 ± 67.50 N for the stroke group and 153.77 ± 70.12 N for the control group.

### Motor Control

On the visuomotor tracking task, the high-functioning stroke group had significantly greater RMSE of ankle position (|*t*_39_| = −3.52; *p* < 0.01; Figure 2C) compared with the control group. The high-functioning stroke group had significantly greater SD of ankle position (|*t*_39_| = −1.77; *p* < 0.05; Figure 2D) compared with the control group.

### Functional Mobility

**Figure 3:**
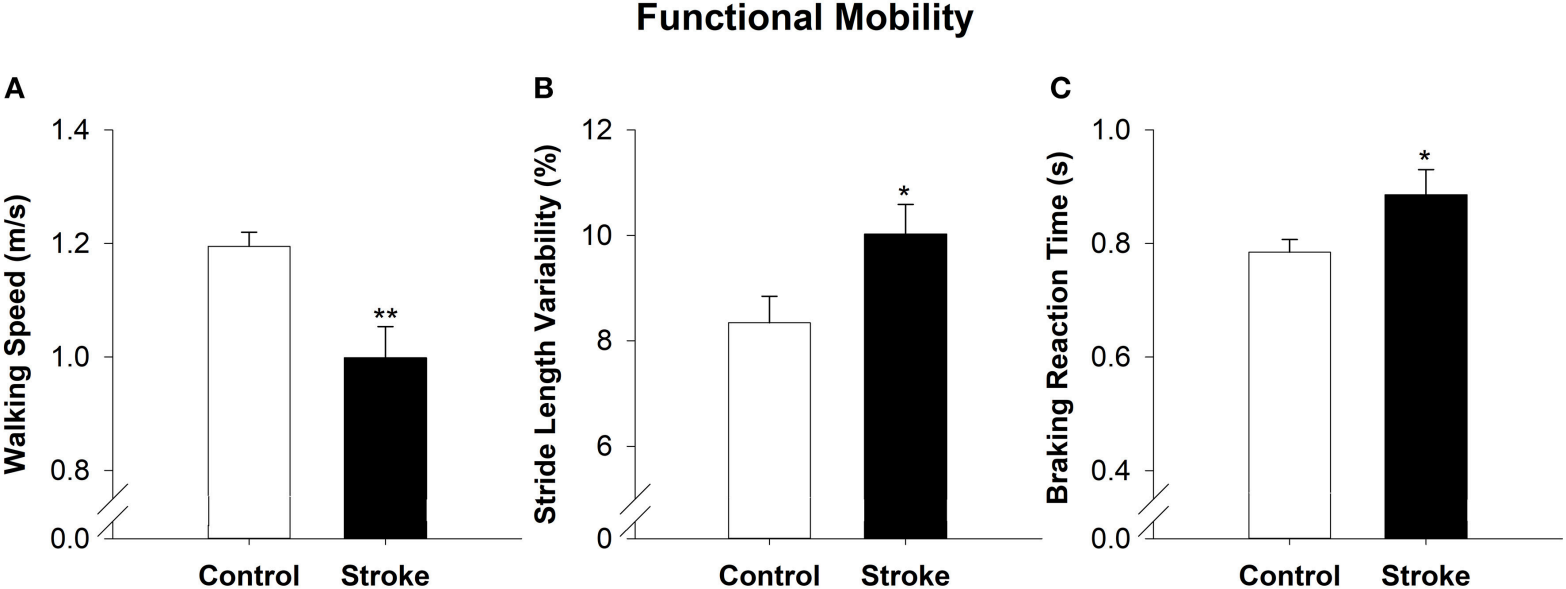
Functional mobility in high-functioning stroke. Functional mobility during overground walking was quantified with walking speed, and **(A)** stride length variability **(B)**. Functional mobility during simulator driving was quantified with braking reaction time **(C)**. The high-functioning stroke group had significantly reduced walking speed, increased stride length variability and increased braking reaction time than the control group. Overall, high-functioning stroke participants demonstrated deficits in functional mobility compared with the healthy controls. *p < 0.05; **p < 0.01.

On the overground walking task, the high-functioning stroke group had significantly reduced walking speed (|*t*_40_| = −3.24; *p* < 0.01; Figure 3A) and increased stride length variability than the control group (|*t*_40_| = −2.22; *p* < 0.05; Figure 3B). During simulated driving, the high-functioning stroke group demonstrated significantly increased braking reaction time compared with the control group (|*t*_39_| = −2.04; *p* < 0.05; Figure 3C).

### Relation Between Strength, Motor Control, Walking, and Driving Function in High-Functioning Stroke

Table 2 shows the correlations between strength, motor control and walking and simulated driving. Motor control measures were significantly correlated with walking speed, stride-length variability and braking reaction time. Specifically, the RMSE was negatively correlated with walking speed (*r* = −0.78, *p* < 0.001), positively correlated with stride length variability (*r* = 0.45, *p* < 0.05) and braking reaction time (*r* = 0.47, *p* < 0.05). The standard deviation was negatively correlated with the walking speed (*r* = −0.69, *p* < 0.01), positively correlated with stride length variability (*r* = 0.58, *p* < 0.05) and braking reaction time (*r* = 0.45, *p* < 0.05). There was no significant correlation between plantarflexion or dorsiflexion strength and walking speed, stride length variability, or braking reaction time (*p* > 0.05). Further, no significant correlations were found between strength and motor control. Specifically, plantarflexion strength was not correlated to RMSE (*r* = −0.45, *p* = 0.85) and SD (*r* = −0.22, *p* = 0.35). Similarly, dorsiflexion strength was not correlated to RMSE (*r* = −0.38, *p* = 0.09) and SD (*r* = −0.35, *p* = 0.13).

**Table 2:** Pearson's Bivariate Correlations (*r*) of strength and motor control measures with walking and driving in stroke.

	**MVC plantarflexion**		**MVC dorsiflexion**		**RMSE**		***SD***	
	***r***	***p***	***r***	***p***	***r***	***p***	***r***	***p***
Walking speed	0.06	0.78	0.34	0.12	**−0.78**	0.00^*^	**-0.69**	0.00^*^
Stride length variability	−0.05	0.82	−0.31	0.18	**0.45**	0.04^*^	**0.58**	0.008^*^
Braking reaction time	−0.12	0.61	−0.28	0.22	**0.47**	0.04^*^	**0.45**	0.05^*^

*MVC, maximum voluntary contraction; RMSE, root mean squared error; SD, standard deviation*,

* *significant. Bold numbers indicate significant correlations*.

### Contribution of Strength and Motor Control to Overground Walking and Driving in High-Functioning Stroke

**Figure 4:**
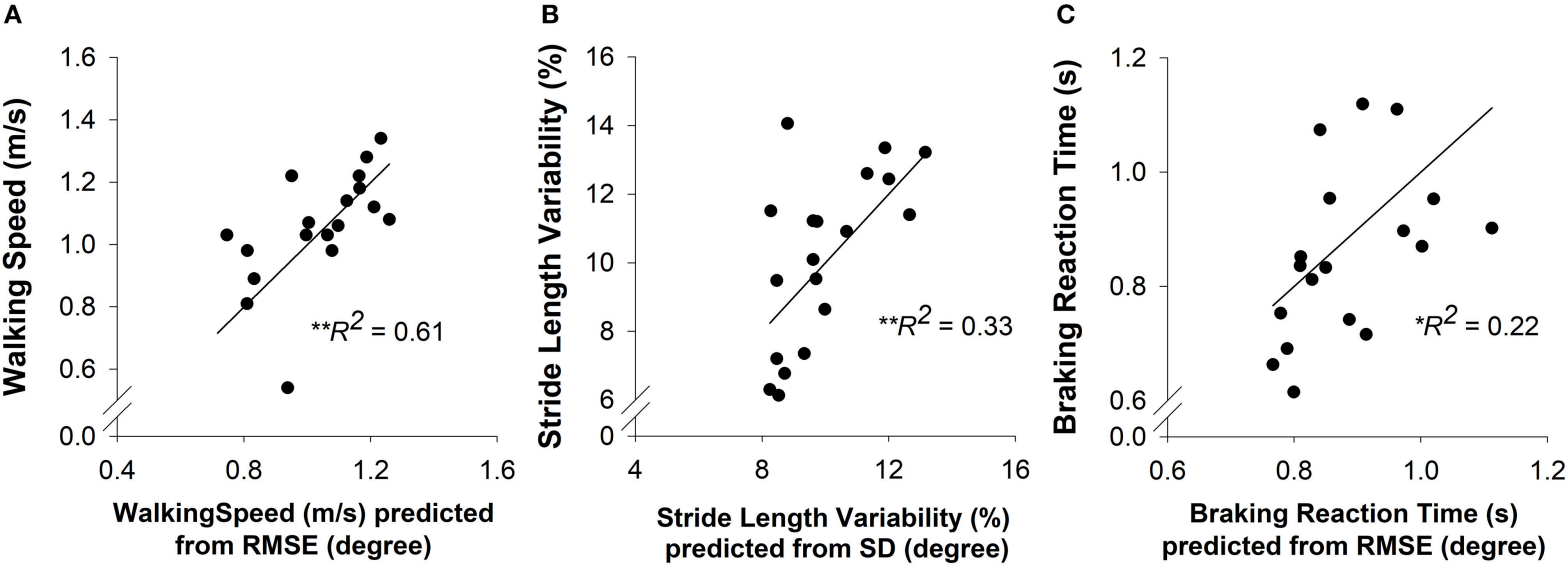
A multiple-linear regression was conducted to predict the overground walking speed, stride length variability, and braking reaction time (criterion variables) from the strength (MVC plantarflexion and MVC dorsiflexion) and motor control (accuracy and variability of ankle position) of each participant (predictor variables). **(A)** Prediction of walking speed. The motor accuracy was a significant predictor (*R*^2^ = 0.61, *p* < 0.01) of walking speed in high-functioning stroke group. **(B)** Prediction of stride length variability. The motor variability was a significant predictor (*R*^2^ = 0.33, *p* < 0.01) of stride length variability in high-functioning stroke group. **(C)** Prediction of braking reaction time. The motor accuracy was a significant predictor (*R*^2^ = 0.22. *p* < 0.05) of braking reaction time in high-functioning stroke group.

To determine the contribution of strength and motor control to overground walking and driving in high-functioning stroke participants, we ran backward multiple-linear regression models. The walking speed was predicted by RMSE of ankle position (*R*^2^ = 0.61, adjusted *R*^2^ = 0.59; *p* < 0.001; Figure 4A). The stride length variability was predicted by SD of ankle position (*R*^2^ = 0.33, adjusted *R*^2^ = 0.29; *p* < 0.01; Figure 4B). The braking reaction time was predicted by RMSE of ankle position (*R*^2^ = 0.22, adjusted *R*^2^ = 0.18; *p* < 0.05; Figure 4C). These regression models indicated that impaired motor control is a predictor of impaired walking and driving performance in high-functioning stroke individuals.

## Discussion

The purpose of this study was to quantify impairments in strength and motor control and determine their contribution to overground walking and driving in high-functioning stroke individuals. Our findings suggest that high-functioning stroke individuals exhibit reduced plantarflexion strength, reduced motor accuracy, and increased motor variability relative to healthy controls. Further, high-functioning stroke individuals demonstrate reduced walking speed, increased stride length variability, and increased braking reaction time compared with healthy controls. The functional deficits in walking and driving were predicted by impairments in motor control (accuracy or variability), but not by strength. Thus, for the first time in the stroke literature we provide evidence that impaired motor control is a significant contributor to functional deficits in high-functioning stroke.

### Motor Control and Strength Deficits in High-Functioning Stroke

High-functioning stroke individuals demonstrated significant decline in ankle motor control. While declines in ankle strength following stroke have been documented extensively, the evidence regarding decline in accuracy and variability of ankle motor control is not well-established (42–44). Few previous studies showed accuracy index during ankle tracking task was impaired in individuals with stroke (13, 41). In our study, high-functioning stroke individuals show decreased motor accuracy (52% more RMSE) and increased motor variability (23% more SD) compared with healthy controls. Decline in motor control has been demonstrated in upper-limb for stroke individuals and is reported to be independent of motor weakness (3, 8, 45). Further, motor control deficits have been studied generally across individuals with varying degree of motor impairments (14, 46). Here, we provide evidence that stroke survivors with a mild to moderate degree of motor impairments demonstrate large and robust differences in accuracy and variability of ankle motor control compared to healthy controls.

High-functioning stroke individuals demonstrate reduced plantarflexion strength (17% less) but no significant differences in dorsiflexion strength compared with healthy controls. Decline in plantarflexion strength is consistent with previous reports of stroke-related weakness in plantarflexors, a key muscle group for forward propulsion during overground walking (40, 47). However, no significant decline in dorsiflexion strength was noted. One reason for this finding could be that the relative decline (paretic compared to non-paretic side) in dorsiflexion strength is presumably less than the relative decline in plantarflexion strength (39). Adams et al., reported that the average degree of weakness was greater for ankle plantarflexors than ankle dorsiflexors. Another possibility is that perhaps the recovery of dorsiflexion strength happens earlier or to greater extent than the recovery of plantarflexion strength. The current findings motivate future investigations to understand whether the relative decline and rate of recovery differs across dorsiflexors and plantarflexors. While these propositions need to be investigated, our findings suggest that high-functioning stroke individuals exhibit declines in plantarflexion but not dorsiflexion strength. Together, these results suggest that ankle strength showed relatively small decline while ankle motor control was significantly impaired in high-functioning stroke individuals.

A recent study showed that strength recovers at a faster rate than motor control in individuals with stroke (48). Our findings extend this work by showing that high-functioning stroke individuals on the upper end of the stroke recovery continuum demonstrate significant deficits in motor control but relatively small strength deficits. These results are important because impaired motor control may contribute to functional deficits in high-functioning stroke individuals and lead to overall reduction in quality of life.

### Functional Deficits in High-Functioning Stroke

Independent walking is a critical recovery goal for high-functioning stroke individuals who often resume pre-stroke activities of daily living such as working and social engagements. Walking speed is a primary determinant of functional ability and social participation (49, 50). In our study, individuals with high-functioning stroke demonstrate reductions in overground walking speed compared to healthy controls. Reduced walking speed indicates incomplete recovery of functional mobility (23, 51). Furthermore, we found that high-functioning stroke individuals demonstrate increased stride length variability as compared with the controls. Increased gait variability is related to poor dynamic balance and highlights the motor system's dysfunction in adapting gait parameters to environmental requirements (52–54). Consequently, increased walking variability may predispose high-functioning stroke individuals to greater risk of falls.

Another important functional task for maintaining independence after stroke is driving. In this study, we examined driving ability in a simulated driving environment by quantifying braking reaction time. Often, safe driving requires the ability to respond to unpredictable events or objects (such as sudden lane change by another car or a child crossing the road) (55). Consequently, braking reaction time is known to be predictor of crash-risk and overall driving ability (56, 57). Our results show that braking reaction time is increased in high-functioning stroke individuals. Our previous work suggests that slower response during reactive driving is linked to increased variability in the motor output and impairs driving function in older adults (58). Increased braking reaction time could result in slower response to dynamically changing traffic conditions and increase the chances for driving accidents in high-functioning stroke individuals. Further, braking reaction time also involves visual and cognitive abilities (59). While all participants in our study reported a 20/20 vision and were able to follow a three-step command, we did not systematically test how specific aspects of visual and cognitive function such as attention, processing speed and decision-making influence braking response time. Future studies are recommended to examine the specific contribution of visual, cognitive, and motor function to braking response after stroke. In summary, high-functioning stroke individuals demonstrate substantial deficits in functional mobility in both overground walking and driving ability.

### Declines in Motor Control Impair Functional Capacity in High-Functioning Stroke

The most interesting finding in this study is that functional deficits in high-functioning stroke are predicted by declines in motor control, but not strength. Our results suggest that motor accuracy is a significant predictor of walking speed and motor variability is a significant predictor of stride length variability. The visuomotor tracking task required participants to accurately and consistently match a sinusoidal target with ankle movements. Perhaps, the inability to accurately and steadily control ankle movement reflects a general inability to modulate the spatio-temporal dynamics of the lower limb resulting in impaired overground walking. Interestingly, we found that overground walking performance was not related to strength deficits in high-functioning stroke individuals. Conventionally, strength is considered to be an indicator of recovery and is correlated to functional capacity in individuals with stroke (60). For example, previous studies have shown that reduced plantarflexion strength limits walking speed in stroke (40, 61). However, previous studies did not apply a stringent criterion to identify and select a specific subgroup of stroke individuals with high-functioning status and allowed the use of assistive devices for some participants during walking. Presumably, with the considerable recovery of ankle strength in high-functioning stroke individuals, strength no longer remained a primary limiting factor in regulation of overground walking speed.

The simulated driving task required the ability to respond as fast as possible to the STOP stimulus while driving in a simulated environment. Our results suggest that the motor accuracy of ankle position predicted the braking reaction time. Stroke-related decrease in motor accuracy may interfere with the ability to react fast, and decrease the speed of response while driving. Another noteworthy point is that driving function after stroke has not be examined in the context of motor impairments before. While, numerous studies have demonstrated that impaired driving in stroke relates to cognitive deficits, the contribution of motor impairments to driving deficits in stroke is largely undetermined (62, 63). Our findings extend previous work by demonstrating that impaired motor control is associated with diminished driving function in high-functioning stroke individuals. Further, ankle strength did not contribute to the braking task perhaps because the driving task did not require individuals to exert maximal force with the ankle, rather to exert submaximal ankle forces to control the gas and the brake pedals.

Together, these findings suggest that following substantial recovery of strength, any further improvement in functional capacity are more reliant on motor control than strength. These findings are in line with two sets of evidence: (1) A recent study that tracked 54 stroke patients over the first year showed that once strength recovers to above 60%, strength and motor control follow separate trajectory of improvement (48). (2) Functional deficits in older adults with minimal weakness were linked to reductions in motor variability but not strength (64, 65). Thus, strength and motor control appear to be independent after a certain strength recovery threshold is met. Further, we found no association between strength and motor control measures. Perhaps, a more complex, non-linear relationship may link force variability and strength (66). However, the independence of strength and motor control in high-functioning stroke individuals in the current study argues that impaired motor control but not strength is a significant contributor to deficits in functional mobility in high-functioning stroke individuals. Currently, strength training interventions have been extensively tested to improve functional capacity after stroke (67, 68). Given the contribution of motor control to functional capacity, training protocols that improve accuracy and variability may augment the recovery of function, especially in high-functioning stroke individuals.

### Considerations

Currently, in the stroke literature, there is no clear definition to distinguish stroke individuals as high or low functioning. Few studies have used the Fugl-Meyer Assessment to classify stroke individuals into severe, moderate, and mild motor impairment categories (69). FMA may be insufficient to gauge a stroke individual's involvement in activities of daily living. Consequently, we determined motor impairment level alone to be inadequate for providing insights into functional status of an individual. In the current study, we identified high-functioning stroke individuals with two distinct criteria-FAI to measure activity level and participation and FMA to determine impairments of body function. Future studies should test the validity of this approach in distinguishing stroke survivors on functional status. Further, we did not compare high-functioning stroke individuals with low-functioning individuals. As stroke individuals regain partial motor abilities, the functional goals escalate progressively. Increased functional independence poses new challenges on the motor system that may require motor capabilities beyond adequate strength alone. Further research is required to determine the contribution of strength and motor control to functional capacity in stroke individuals with varying degrees of functional status.

Another issue concerns visuomotor tracking approach for understanding the contribution of motor control to walking, Visuomotor tracking provides a simple approach for tapping into neuromuscular system's ability to integrate and modulate motor output for performing functional mobility tasks. Specifically, the visuomotor tracking involves three functional domains (a) attentional resources (70, 71), (b) visual processing to transform the information about target position into movement planning (72), and (c) motor ability to produce steady and accurate movements (8, 73). Similarly, walking requires the complex integration of cognitive, visual, and motor functions (74, 75). Therefore, visumotor-tracking provides a model task for understanding the neuromotor control of movement involved in walking. While this approach has been used to study posture and gait in individuals with Parkinson's disease (76), future research is needed to test the validity of this approach in stroke. In addition, the participant positioning for strength and motor control measurements corresponded better with simulated driving than the walking task. Futures studies should examine these measures in upright positions to understand the contributions of strength and motor control to walking. Finally, whether current findings from simulated driving environment extend to on-road driving after stroke needs to be tested.

## Conclusion

We provide novel evidence that declines in motor control and not strength impair functional capacity in high-functioning stroke survivors. We demonstrated that although high-functioning stroke individuals show substantial recovery of strength, they continue to show impairments in motor control and functional mobility. Most importantly, functional deficits in walking and driving were related to decline in motor control but not strength. Rehabilitation interventions assessing and improving motor control will potentially enhance functional outcomes in high-functioning stroke survivors.

## Ethics Statement

This study was carried out in accordance with the recommendations of office of Human Research protection of University of Florida with written informed consent from all subjects. All subjects gave written informed consent in accordance with the Declaration of Helsinki. The protocol was approved by the Institution Review Board of University of Florida.

## Author Contributions

NL and EC conceived and designed the experiments. NL and PP conducted data analysis, data interpretation, and drafted the manuscript. NL, AC-M, and EH conducted data collection. NL, PP, AC-M, EH, SP, and EC revised and approved the manuscript for publication.

### Conflict of Interest Statement

The authors declare that the research was conducted in the absence of any commercial or financial relationships that could be construed as a potential conflict of interest.

## References

[1] Patten C Lexell J Brown HE. Weakness and strength training in persons with poststroke hemiplegia: rationale, method, and efficacy. J Rehabil Res Dev. (2004) 41:293–312. 10.1682/JRRD.2004.03.0293 15543447

[2] Lang CE Bland MD Bailey RR Schaefer SY Birkenmeier RL. Assessment of upper extremity impairment, function, and activity after stroke: foundations for clinical decision making. J Hand Ther. (2013) 26:104–14. 10.1016/j.jht.2012.06.005 22975740 PMC3524381

[3] Kang N Cauraugh JH. Force control in chronic stroke. Neurosci Biobehav Rev. (2015) 52:38–48. 10.1016/j.neubiorev.2015.02.005 25704075

[4] Bourbonnais D Vanden Noven S. Weakness in patients with hemiparesis. Am J Occup Ther. (1989) 43:313–9. 2655457 10.5014/ajot.43.5.313

[5] Tyson SF Chillala J Hanley M Selley AB Tallis RC. Distribution of weakness in the upper and lower limbs post-stroke. Disabil Rehabil. (2006) 28:715–9. 10.1080/09638280500301584 16809214

[6] Cirstea MC Levin MF. Compensatory strategies for reaching in stroke. Brain (2000) 123:940–53. 10.1093/brain/123.5.940 10775539

[7] Naik SK Patten C Lodha N Coombes SA Cauraugh JH. Force control deficits in chronic stroke: grip formation and release phases. Exp Brain Res. (2011) 211:1–15. 10.1007/s00221-011-2637-8 21448576

[8] Lindberg PG Roche N Robertson J Roby-Brami A Bussel B Maier MA. Affected and unaffected quantitative aspects of grip force control in hemiparetic patients after stroke. Brain Res. (2012) 1452:96–107. 10.1016/j.brainres.2012.03.007 22464180

[9] Allgower K Hermsdorfer J Fine motor skills predict performance in the jebsen taylor hand function test after stroke. Clin Neurophysiol. (2017) 28:1858–71. 10.1016/j.clinph.2017.07.408 28826016

[10] Marigold DS Eng JJ Tokuno CD Donnelly CA. Contribution of muscle strength and integration of afferent input to postural instability in persons with stroke. Neurorehabil Neural Repair. (2004) 18:222–9. 10.1177/1545968304271171 15537993 PMC3226790

[11] Nakamura R Hosokawa T Tsuji I. Relationship of muscle strength for knee extension to walking capacity in patients with spastic hemiparesis. Tohoku J Exp Med. (1985) 145:335–40. 4002220 10.1620/tjem.145.335

[12] Bohannon RW Andrews AW. Correlation of knee extensor muscle torque and spasticity with gait speed in patients with stroke. Arch Phys Med Rehabil. (1990) 71:330–3. 2327887

[13] Carey JR Anderson KM Kimberley TJ Lewis SM Auerbach EJ Ugurbil K. fMRI analysis of ankle movement tracking training in subject with stroke. Exp Brain Res. (2004) 154:281–90. 10.1007/s00221-003-1662-7 14578998

[14] Chow JW Stokic DS. Force control of quadriceps muscle is bilaterally impaired in subacute stroke. J Appl Physiol. (2011)111:1290–1295. 10.1152/japplphysiol.00462.2011 21885803

[15] Kim Y Kim WS Koh K Yoon B Damiano DL Shim JK. Deficits in motor abilities for multi-finger force control in hemiparetic stroke survivors. Exp Brain Res. (2016) 234:2391–402. 10.1007/s00221-016-4644-2 27071926

[16] Buchner DM Beresford SA Larson EB LaCroix AZ Wagner EH Effects of physical activity on health status in older adults. II Intervention studies. Annu Rev Public Health (1992) 13:469–88. 10.1146/annurev.pu.13.050192.002345 1599599

[17] Jorgensen HS Nakayama H Raaschou HO Vive-Larsen J Stoier M Olsen TS Outcome and time course of recovery in stroke. Part II: Time course of recovery The Copenhagen Stroke Study. Arch Phys Med Rehabil. (1995) 76:406–12. 7741609 10.1016/s0003-9993(95)80568-0

[18] Jorgensen HS Nakayama H Raaschou HO Vive-Larsen J Stoier M Olsen TS Outcome and time course of recovery in stroke. part I: outcome the copenhagen stroke study. Arch Phys Med Rehabil. (1995) 76:399–405. 7741608 10.1016/s0003-9993(95)80567-2

[19] Jorgensen HS Nakayama H Raaschou HO Olsen TS. Recovery of walking function in stroke patients: the Copenhagen Stroke Study. Arch Phys Med Rehabil. (1995) 76:27–32. 7811170 10.1016/s0003-9993(95)80038-7

[20] Smith MC Byblow WD Barber PA Stinear CM. Proportional recovery from lower limb motor impairment after stroke. Stroke (2017) 48:1400–3. 10.1161/strokeaha.116.016478 28341754

[21] Busch MA Coshall C Heuschmann PU McKevitt C Wolfe CD. Sociodemographic differences in return to work after stroke: the South London Stroke Register (SLSR). J Neurol Neurosurg Psychiatry (2009) 80:888–93. 10.1136/jnnp.2008.163295 19276102

[22] van Velzen JM van Bennekom CA Edelaar MJ Sluiter JK Frings-Dresen MH. How many people return to work after acquired brain injury?: a systematic review. Brain Inj. (2009) 23:473–88. 10.1080/02699050902970737 19484621

[23] Perry J Garrett M Gronley JK Mulroy SJ. Classification of walking handicap in the stroke population. Stroke (1995) 26:982–9. 7762050 10.1161/01.str.26.6.982

[24] Lord SE McPherson K McNaughton HK Rochester L Weatherall M. Community ambulation after stroke: how important and obtainable is it and what measures appear predictive? Arch Phys Med Rehabil. (2004) 85:234–9. 10.1016/j.apmr.2003.05.002 14966707

[25] van de Port IG Kwakkel G Lindeman E. Community ambulation in patients with chronic stroke: how is it related to gait speed? J Rehabil Med. (2008) 40:23–7. 10.2340/16501977-0114 18176733

[26] Nakamura T Meguro K Sasaki H. Relationship between falls and stride length variability in senile dementia of the Alzheimer type. Gerontology (1996) 42:108–13. 10.1159/000213780 9138973

[27] Hausdorff JM Rios DA Edelberg HK. Gait variability and fall risk in community-living older adults: a 1-year prospective study. Arch Phys Med Rehabil. (2001) 82:1050–6. 10.1053/apmr.2001.24893 11494184

[28] Mirelman A Herman T Brozgol M Dorfman M Sprecher E Schweiger A. Executive function and falls in older adults: new findings from a five-year prospective study link fall risk to cognition. PLoS ONE (2012) 7:e40297. 10.1371/journal.pone.0040297 22768271 PMC3386974

[29] Akinwuntan AE Wachtel J Rosen PN. Driving simulation for evaluation and rehabilitation of driving after stroke. J Stroke Cerebrovasc Dis. (2012) 21:478–86. 10.1016/j.jstrokecerebrovasdis.2010.12.001 21236698

[30] Sagberg F Bjornskau T. Hazard perception and driving experience among novice drivers. Acc Anal Prev. (2006) 38:407–14. 10.1016/j.aap.2005.10.014 16313881

[31] Jurecki RS Stanczyk TL Driver reaction time to lateral entering pedestrian in a simulated crash traffic situation. transportation research Part F-traffic. Psychol. Behav. (2014) 27:22–36. 10.1016/j.trf.2014.08.006

[32] Duncan PW Lai SM Keighley J. Defining post-stroke recovery: implications for design and interpretation of drug trials. Neuropharmacology (2000) 39:835–41. 10.1016/S0028-3908(00)00003-4 10699448

[33] Patel M Tilling K Lawrence E Rudd A Wolfe C McKevitt C. Relationships between long-term stroke disability, handicap and health-related quality of life. Age Ageing (2006) 35:273–9. 10.1093/ageing/afj074 16638767

[34] van Wijk I Lindeman E Kappelle L van Gijn J Koudstaal PJ Gorter JW Functional status and use of healthcare facilities in long-term survivors of transient ischaemic attack or minor ischaemic stroke. J. Neurol. Neurosurg. Psychiatry (2006) 77:1238–43. 10.1136/jnnp.2006.089391 16735396 PMC2077397

[35] Gladstone DJ Danells CJ Black SE. The fugl-meyer assessment of motor recovery after stroke: a critical review of its measurement properties. Neurorehabil Neural Repair. (2002) 16:232–40. 10.1177/154596802401105171 12234086

[36] Schuling J de Haan R Limburg M Groenier KH. The Frenchay activities index. Assessment of functional status in stroke patients Stroke (1993) 24:1173–7. 8342192 10.1161/01.str.24.8.1173

[37] Kendall FP McCreary FK Provance PG Rodgers MM Romani WA Fundamental concepts. In: Lappies P Seitz A editors, Muscles: Testing and Function, with Posture and Pain. 5th ed. (Baltimore, MD: Lippincott Williams & Wilkins) (2005), p. 14–7.

[38] Magee DJ Lower leg, ankle, and foot. In: Falk K editor. Orthopedic Physical Assessment. St. Louis, MO: Elsevier Inc (2014), p. 920.

[39] Adams RW Gandevia SC Skuse NF. The distribution of muscle weakness in upper motoneuron lesions affecting the lower limb. Brain (1990) 113:1459–76. 2245306 10.1093/brain/113.5.1459

[40] Nadeau S Gravel D Arsenault AB Bourbonnais D. Plantarflexor weakness as a limiting factor of gait speed in stroke subjects and the compensating role of hip flexors. Clin Biomech. (1999) 14:125–35. 10619100 10.1016/s0268-0033(98)00062-x

[41] Madhavan S Weber KA II Stinear JW. Non-invasive brain stimulation enhances fine motor control of the hemiparetic ankle: implications for rehabilitation. Exp Brain Res. (2011) 209:9–17. 10.1007/s00221-010-2511-0 21170708

[42] Bohannon RW Strength of lower limb related to gait velocity and cadence in stroke patients. Physiotherapy Canada (1986) 38:204–6.

[43] Andrews AW Bohannon RW. Distribution of muscle strength impairments following stroke. Clin Rehabil. (2000) 14:79–87. 10.1191/026921500673950113 10688348

[44] Pohl PS Startzell JK Duncan PW Wallace D. Reliability of lower extremity isokinetic strength testing in adults with stroke. Clin Rehabil. (2000) 14:601–7. 10.1191/0269215500cr367oa 11128734

[45] Archer DB Kang N Misra G Marble S Patten C Coombes SA. Visual feedback alters force control and functional activity in the visuomotor network after stroke. Neuroimage Clin. (2018) 17:505–17. 10.1016/j.nicl.2017.11.012 29201639 PMC5700823

[46] Lodha N Naik SK Coombes SA Cauraugh JH. Force control and degree of motor impairments in chronic stroke. Clin Neurophysiol. (2010) 121:1952–61. 10.1016/j.clinph.2010.04.005 20435515

[47] Klein CS Brooks D Richardson D McIlroy WE Bayley MT. Voluntary activation failure contributes more to plantar flexor weakness than antagonist coactivation and muscle atrophy in chronic stroke survivors. J Appl Physiol. (2010) 109:1337–46. 10.1152/japplphysiol.00804.2009 20724561

[48] Xu J Ejaz N Hertler B Branscheidt M Widmer M Faria AV. Separable systems for recovery of finger strength and control after stroke. J Neurophysiol. (2017) 118:1151–63. 10.1152/jn.00123.2017 28566461 PMC5547267

[49] Fulk GD Reynolds C Mondal S Deutsch JE. Predicting home and community walking activity in people with stroke. Arch Phys Med Rehabil. (2010) 91:1582–6. 10.1016/j.apmr.2010.07.005 20875518

[50] Jalayondeja C Kaewkungwal J Sullivan PE Nidhinandana S Pichaiyongwongdee S Jareinpituk S. Factors related to community participation by stroke victims six month post-stroke. Southeast Asian J Trop Med Public Health (2011) 42:1005–13. 22299484

[51] Amatachaya S Chuadthong J Thaweewannaku T Srisim K Phonthee S. Levels of community ambulation ability in patients with stroke who live in a rural area. Malays J Med Sci. (2016) 23:56–62. 27540326 PMC4975589

[52] Schaafsma JD Giladi N Balash Y Bartels AL Gurevich T Hausdorff JM. Gait dynamics in Parkinson's disease: relationship to Parkinsonian features, falls and response to levodopa. J Neurol Sci. (2003) 212:47–53. 10.1016/S0022-510X(03)00104-7 12809998

[53] Terrier P Schutz Y. Variability of gait patterns during unconstrained walking assessed by satellite positioning (GPS). Eur J Appl Physiol. (2003) 90:554–61. 10.1007/s00421-003-0906-3 12905048

[54] Hausdorff JM. Gait dynamics, fractals and falls: finding meaning in the stride-to-stride fluctuations of human walking. Hum Mov Sci. (2007) 26:555–89. 10.1016/j.humov.2007.05.003 17618701 PMC2267927

[55] Anstey KJ Wood J Lord S Walker JG. Cognitive, sensory and physical factors enabling driving safety in older adults. Clin Psychol Rev. (2005) 25:45–65. 10.1016/j.cpr.2004.07.008 15596080

[56] Summala H Brake reaction times and driver behavior analysis. Transport. Hum. Fact. (2000) 2:217–26. 10.1207/STHF0203_2

[57] Li Y Zheng Y Wang J Kodaka K Li K. Crash probability estimation via quantifying driver hazard perception. Accid Anal Prev. (2018) 116:116–25. 10.1016/j.aap.2017.05.009 28595973

[58] Lodha N Moon H Kim C Onushko T Christou EA. Motor output variability impairs driving ability in older adults. J Gerontol A Biol Sci Med Sci. (2016) 71:1676–81. 10.1093/gerona/glw013 26935111

[59] Hird MA Vetivelu A Saposnik G Schweizer TA. Cognitive, on-road, and simulator-based driving assessment after stroke. J Stroke Cerebrovasc Dis. (2014) 23:2654–70. 10.1016/j.jstrokecerebrovasdis.2014.06.010 25306401

[60] Sunderland A Tinson D Bradley L Hewer RL. Arm function after stroke. An evaluation of grip strength as a measure of recovery and a prognostic indicator. J Neurol Neurosurg Psychiatry (1989) 52:1267–72. 2592969 10.1136/jnnp.52.11.1267 PMC1031635

[61] Kim CM Eng JJ. The relationship of lower-extremity muscle torque to locomotor performance in people with stroke. Phys Ther. (2003) 83:49–57. 10.1093/ptj/83.1.49 12495412

[62] Nouri FM Tinson DJ Lincoln NB. Cognitive ability and driving after stroke. Int Disabil Stud. (1987) 9:110–5. 3429395 10.3109/03790798709166334

[63] Marshall SC Molnar F Man-Son-Hing M Blair R Brosseau L Finestone HM. Predictors of driving ability following stroke: a systematic review. Top Stroke Rehabil. (2007) 14:98–114. 10.1310/tsr1401-98 17311796

[64] Christou EA Yang Y Rosengren KS. Taiji training improves knee extensor strength and force control in older adults. J Gerontol A Biol Sci Med Sci. (2003) 58:763–6. 10.1093/gerona/58.8.M763 12902537

[65] Marmon AR Gould JR Enoka RM. Practicing a functional task improves steadiness with hand muscles in older adults. Med Sci Sports Exerc. (2011) 43:1531–7. 10.1249/MSS.0b013e3182100439 21266932

[66] Hamilton AF Jones KE Wolpert DM. The scaling of motor noise with muscle strength and motor unit number in humans. Exp Brain Res. (2004) 157:417–30. 10.1007/s00221-004-1856-7 15014922

[67] Weiss A Suzuki T Bean J Fielding RA. High intensity strength training improves strength and functional performance after stroke. Am J Phys Med Rehabil. (2000) 79:391–64. 10.1097/00002060-200007000-00009 10892623

[68] Ada L Dorsch S Canning CG. Strengthening interventions increase strength and improve activity after stroke: a systematic review. Aust J Physiother. (2006) 52:241–8. 10.1016/S0004-9514(06)70003-4 17132118

[69] Hoonhorst MH Nijland RH van den Berg JS Emmelot CH Kollen BJ Kwakkel G. How do fugl-meyer arm motor scores relate to dexterity according to the action research arm test at 6 months poststroke? Arch Phys Med Rehabil. (2015) 96:1845–9. 10.1016/j.apmr.2015.06.009 26143054

[70] Hocherman S Moont R Schwartz M. Recruitment of attentional resources during visuomotor tracking: effects of Parkinson's disease and age. Brain Res Cogn Brain Res. (2004) 21:77–86. 10.1016/j.cogbrainres.2004.05.008 15325415

[71] Seidler RD Kwak Y Fling BW Bernard JA Neurocognitive mechanisms of error-based motor learning In: Richardson MJ Riley MA Shockley K. editors. Progress in Motor Control: Neural, Computational and Dynamic Approaches. New York, NY: Springer (2013).

[72] Grafton ST Schmitt P Van Horn J Diedrichsen J. Neural substrates of visuomotor learning based on improved feedback control and prediction. Neuroimage (2008) 39:1383–95. 10.1016/j.neuroimage.2007.09.062 18032069 PMC2268716

[73] Seitz RJ Kammerzell K Samartzi M Jander S Wojtecki L Verschure PFMJ Monitoring of visuomotor coordination in healthy subjects and patients with stroke and parkinson's disease: an application study using the PABLOR-Device. Int J Neurorehabilitation. (2014) 1:113 10.4172/2376-0281.1000113

[74] Drew T Andujar JE Lajoie K Yakovenko S. Cortical mechanisms involved in visuomotor coordination during precision walking. Brain Res Rev. (2008) 57:199–211. 10.1016/j.brainresrev.2007.07.017 17935789

[75] Patel P Lamar M Bhatt T. Effect of type of cognitive task and walking speed on cognitive-motor interference during dual-task walking. Neuroscience (2014) 260:140–8. 10.1016/j.neuroscience.2013.12.016 24345478

[76] Inzelberg R Schechtman E Hocherman S. Visuo-motor coordination deficits and motor impairments in Parkinson's disease. PLoS ONE (2008) 3:e3663. 10.1371/journal.pone.0003663 18987752 PMC2576439

